# Matrix Control of Solvent and Electron Flow in a Nonheme Diiron Nitrite Reductase

**DOI:** 10.1021/jacsau.6c00043

**Published:** 2026-03-26

**Authors:** Hung-Ying Chen, Yi-Shan Lu, Chu-Chun Cheng, Feng-Chun Lo, Tzuhsiung Yang, Yun-Wei Chiang

**Affiliations:** Department of Chemistry, 34881 National Tsing Hua University, Hsinchu 300-044, Taiwan

**Keywords:** nonheme diiron enzyme, nitrite reductase, solvent gating, solvent kinetic isotope effect, EPR spectroscopy

## Abstract

Nonheme diiron enzymes catalyze a wide range of biologically essential redox transformations. Despite extensive study, how the surrounding protein matrix coordinates electron transfer, proton delivery, and solvent access and whether mechanistic insights derived *in vitro* reflect enzyme function in living systems remain incompletely understood. Here, we investigate the bacterial enzyme ScdA, a nonheme diiron nitrite reductase that converts nitrite to nitric oxide (NO), to define how first- and second-sphere interactions regulate catalysis. Using site-directed mutagenesis, steady-state kinetics, EPR spectroscopy, solvent kinetic isotope effect analysis, molecular dynamics simulations, and cell-based spin trapping, we identify distinct functional contributions of residues surrounding the diiron center. First-sphere ligands ensure cofactor assembly and redox integrity, whereas second-sphere residues modulate turnover by controlling hydrogen-bond networks and solvent accessibility near the catalytic core. Structural and kinetic analyses reveal a solvent-accessible pathway whose gating properties tune hydration dynamics and influence the rate-limiting step. Importantly, cell-based EPR detection of NO demonstrates that the same structural determinants governing catalytic efficiency *in vitro* also operate under cellular conditions. Together, these results establish controlled hydration as a general design principle in nonheme diiron enzymes.

## Graphical abstract

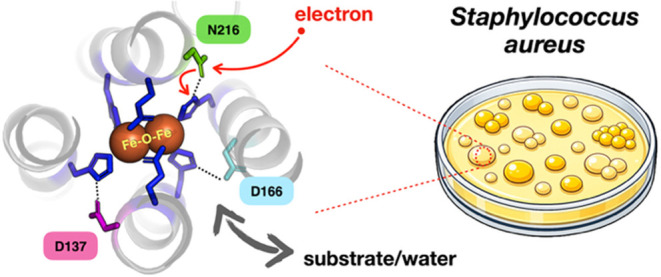

## Introduction

Nonheme diiron enzymes constitute a versatile class of metalloenzymes that catalyze a broad spectrum of oxidative and reductive transformations essential to biological metabolism, including hydrocarbon oxidation, oxygen activation, and nitrogen-oxide interconversion.^1–3^ Their catalytic power derives from a diiron center that can access multiple redox states—including diferrous, mixed-valent (Fe­(II)/Fe­(III)), and diferric forms—to support multielectron redox chemistry.^4–6^ The population and interconversion of these states are further modulated by the protein environment, which tunes electron transfer, proton delivery, and solvent access to enable distinct chemistries across the diiron enzyme family. The diversity of reactions mediated by this family—exemplified by soluble methane monooxygenase (sMMO), AurF, and ribonucleotide reductase (RNR)—has made nonheme diiron enzymes archetypal systems for probing proton-coupled electron transfer and metal-centered reactivity.^7–10^ Despite decades of biochemical, spectroscopic, and computational study, however, how the surrounding protein matrix modulates reactivity, substrate specificity, and solvent access at diiron centers remains incompletely understood.

The bacterial protein ScdA, a recently identified nonheme diiron nitrite reductase from *Staphylococcus aureus*, provides a compelling platform for addressing this problem.^11^ ScdA catalyzes the one-electron reduction of nitrite (NO_2_^–^) to nitric oxide (NO), which is a key step in microbial nitrogen metabolism. Its diiron center is coordinated by a conserved 4-His/2-Glu motif and adopts the canonical fold of the nonheme diiron superfamily. Unlike oxygen-activating diiron enzymes, ScdA operates under anaerobic conditions and can accept electrons from small artificial donors such as methyl viologen *in vitro*.^11^ This feature enables detailed kinetic and mechanistic interrogation, while offering a tractable proxy for physiological electron transfer processes. Although crystallographic studies have defined the first-sphere coordination environment, the roles of nearby second-sphere residues (those shaping hydrogen-bond networks, electrostatics, and solvent accessibility) remain poorly characterized.

More broadly, while second-sphere interactions have been implicated in tuning redox properties and proton delivery in several nonheme diiron enzymes,^12,13^ direct experimental connections between these features and catalytic turnover are limited, particularly for reductive transformations such as nitrite reduction. As a result, a fundamental mechanistic question persists: how does the protein matrix coordinate electron transfer, proton management, and substrate access around a diiron center to sustain efficient catalysis? Equally unresolved is whether mechanistic principles derived from *in vitro* studies accurately describe the enzyme behavior under cellular conditions.

To address these questions, we examined how the first- and second-sphere environments collectively govern the structure, metal loading, redox chemistry, and nitrite-reduction activity of ScdA. Using an integrated approach that combines site-directed mutagenesis, steady-state kinetics, iron quantitation, electron paramagnetic resonance (EPR) spectroscopy, solvent kinetic isotope effect (KIE) analysis, molecular dynamics (MD) simulations, and cell-based spin-trapping measurements of NO production, we define a mechanistic framework linking cofactor integrity with controlled solvent accessibility. By demonstrating that the same structural determinants regulate catalysis both *in vitro* and in living cells, this work bridges chemical mechanism and physiological function, establishing hydration control and solvent gating as central elements of nonheme diiron enzymology.

## Results

### First-Sphere Integrity Is Essential for Nitrite Reduction

We began with the first coordination sphere to establish a baseline requirement for catalysis. The ScdA crystal structure (PDB: 9j47) indicates a diiron center ligated by four histidines (H87, H132, H167, and H211) and two glutamates (E136, E215).^11^ To test whether any single ligand is dispensable, we individually replaced each with leucine and monitored nitrite reductase activity using a methyl viologen (MV) consumption assay (Figure 1A,B), a well-established approach previously demonstrated to faithfully report the nitrite-to-NO conversion catalyzed by ScdA and related nonheme diiron proteins.^11,14^ In this coupled readout, oxidation of the MV radical cation reports directly on nitrite reduction at a 1:1 stoichiometric ratio, so the decay of absorbance at 600 nm quantifies catalytic turnover. Under rigorously anaerobic conditions, WT ScdA (1 μM) mixed with excess MV (enzyme:MV = 1:116) consumed MV robustly, whereas each single first-sphere variant showed negligible MV oxidation (Figure 1B).

**Figure 1:**
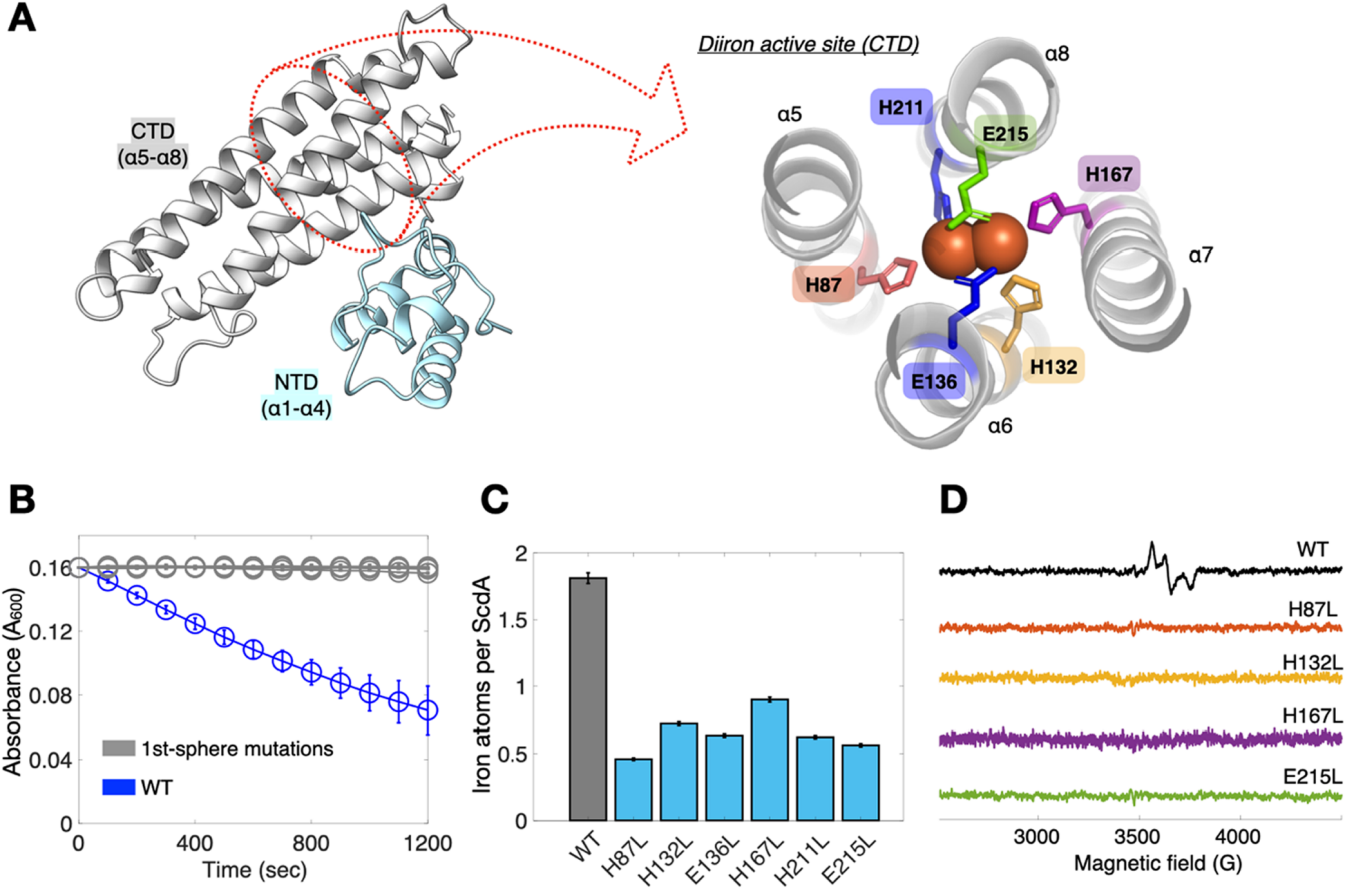
First-sphere residues are essential for diiron-center assembly and nitrite-reduction activity in ScdA. (A) Overall architecture of ScdA showing the N-terminal domain (NTD; α1−α4, cyan) and C-terminal domain (CTD; α5−α8, gray). The diiron center (orange spheres) resides in the CTD and is coordinated by four histidines (H87, H132, H167, H211) and two glutamates (E136, E215). The right panel magnifies the active site, highlighting the first-sphere ligands. (B) MV consumption assay under anaerobic conditions showing time-dependent decay of A_600_. WT (blue) exhibits robust MV oxidation, whereas first-sphere variants (gray) are inactive. (C) Iron quantitation by TPTZ assay. ScdA WT binds ∼1.8 Fe per monomer, while all first-sphere variants display severely reduced iron loading. (D) X-band EPR spectra (10 K) of WT and representative first-sphere variants shown as a qualitative diagnostic of cofactor integrity. WT exhibits the rhombic signal (*g* ≈ 1.96, 1.92, 1.87) characteristic of a mixed-valent diiron center, whereas first-sphere variants are EPR-silent, consistent with loss of the functional cofactor; signal intensities are not used here to quantify mixed-valent populations (see our recent publication for redox-dependent spectroscopic characterization of WT^11^).

To determine why the activity collapsed, we next asked whether cofactor incorporation was compromised. TPTZ assays revealed that all first-sphere variants carried very low iron (≤∼0.6 Fe per ScdA protomer), in contrast to WT (∼1.8 Fe per protomer; Figure 1C).^11,15,16^ EPR spectra at 10 K provided an electronic signature for this defect: WT displayed the characteristic mixed-valent diiron signal (*g* ≈ 1.96, 1.92, 1.87), while representative variants (H87L, H132L, H167L, E215L) were EPR-silent (Figure 1D). These results establish the premise for the remainder of the study: intact first-sphere ligation is essential for the assembly of the diiron center and enabling catalysis.

### Second-Sphere Control by Hydrogen Bonding and Sterics

With the first-sphere requirement defined, we next asked whether residues beyond the metal ligands can tune activity without perturbing metal occupancy—specifically through hydrogen bonding and local electrostatics that couple to electron/proton transfer. Guided by inter-residue distances in the structure, we selected D137, D166, and N216, each positioned to form hydrogen bonds with nearby first-sphere histidines (Figure 2A). For each site, we introduced substitutions spanning hydrogen-bonding capacity, polarity, and side-chain volume, quantified nitrite-reduction specific activity (MV oxidized per min per mg of protein), and measured iron loading in parallel (TPTZ assay).

**Figure 2:**
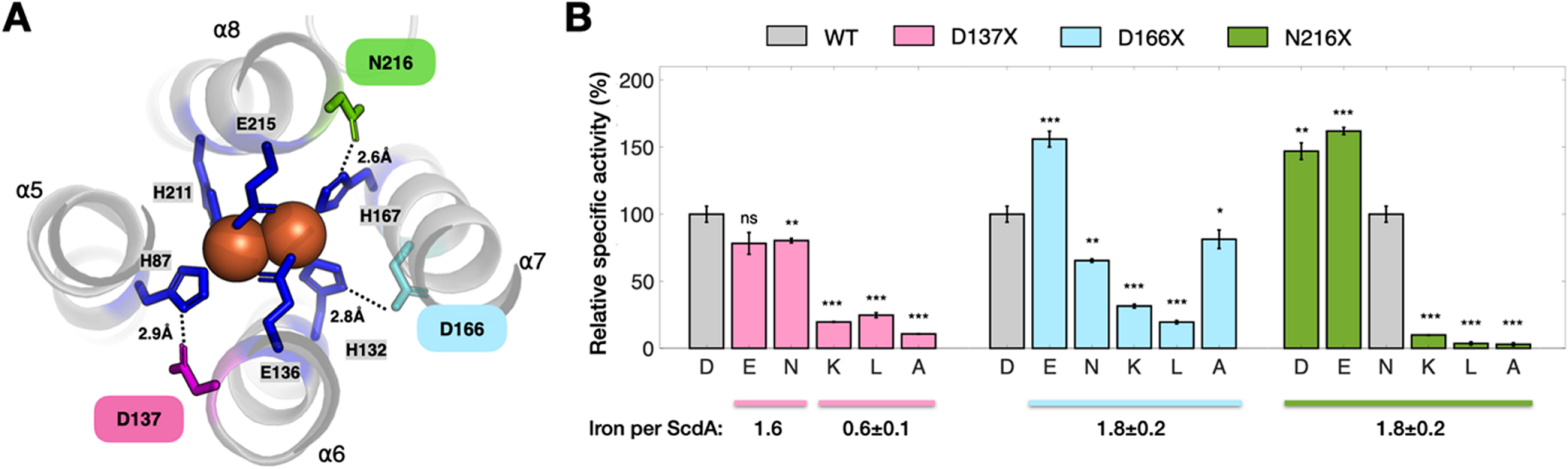
Functional roles of second-sphere residues in modulating ScdA activity. (A) Structural representation of the diiron center and nearby second-sphere residues based on the ScdA crystal structure (PDB 9j47). The diiron cofactor (orange spheres) is coordinated by first-sphere ligands (blue), while D137 (magenta), D166 (cyan), and N216 (green) reside in the second coordination sphere. Dashed lines indicate inter-residue distances to first-sphere histidines, suggesting potential hydrogen-bonding or electrostatic interactions that influence catalysis. (B) Relative specific activities of ScdA variants containing single substitutions at D137, D166, or N216, measured by the MV consumption assay and normalized to WT. Bars represent means ± s.d. of triplicate measurements. Statistical significance versus WT: *p < 0.05, **p < 0.01, ***p < 0.001*; ns*, not significant. Mutations at D137 that disrupt hydrogen bonding (K, L, A) lead to reduced iron loading (∼0.6 ± 0.1 Fe per monomer) and near-complete loss of activity. In contrast, substitutions at D166 and N216 alter activity without changing iron content (∼1.8 ± 0.2 Fe per monomer). See also Figures S1 and S2.

#### D137

D137E and D137N retained activities only slightly below those of WT (Figures 2B and S1) and maintained WT-like iron (∼1.8 Fe per protomer; Figure S2). By contrast, D137 K and D137L dropped to ∼20% of WT, and D137A to ∼10%, each accompanied by a marked iron loss (∼0.6 Fe per protomer). Thus, hydrogen-bonding at D137 appears critical to preserve the local architecture of the diiron site; when lost, iron occupancy and activity decline together.

#### D166

At position D166, we observed a qualitatively different behavior (Figures 2B and S1). All substitutions tested (D166E, D166N, D166K, D166L, and D166A) retained near-WT levels of iron loading (Figure S2), demonstrating that this residue does not play an important role in diiron cofactor assembly or stability. Despite comparable metal incorporation, catalytic activities varied widely (D166E ∼ 150% WT; D166N ∼ 70% WT; D166K ∼ 30% WT; D166L ∼ 20% WT; D166A ∼ 80% WT), indicating that changes at this position primarily affect turnover rather than cofactor formation. Importantly, these activity differences do not scale with hydrogen-bonding capacity or side-chain polarity: both hydrogen-bond-competent (E, N) and nonpolar or charged substitutions (A, L, and K) produce disparate effects (Figure 2B). This lack of a simple structure–activity relationship suggests that D166 does not participate directly in catalysis through specific chemical interactions. Instead, the data are most consistent with a steric and electrostatic gating role, in which the size and charge of the side chain appear to regulate access of substrate and solvent to the diiron center, thereby modulating catalytic efficiency without altering cofactor integrity. On this basis, we, in a later section, examined whether D166 defines or controls an internal access pathway leading to the active site.

#### N216

Substituting N216 with acidic residues (N216D/E) enhanced activity to ∼150% of WT, whereas N216 K/L/A nearly abolished activity (<10% of WT) (Figures 2B and S1). Across all N216 variants, iron loading remained WT-like (∼1.8 Fe per protomer; Figure S2), indicating that this site does not control the cofactor assembly. These data point to hydrogen-bonding capacity—and, by extension, the local electrostatic/proton-coupling network—at position 216 as a primary determinant of catalytic efficiency. Disrupting hydrogen bonding (with N216 K/L/A) at this locus severely compromises nitrite reduction. Among the three second-sphere positions examined, N216 appears to exert the strongest functional control on electron delivery to the diiron core without perturbing metal occupancy.

### A Single Solvent-Accessible Tunnel Positions D166 at the Surface Opening

The D166 variants show substantial changes in activity with minimal effects on iron loading (Figure 2), suggesting that this residue influences substrate or proton access rather than cofactor assembly. We therefore asked whether the crystal structure contains a continuous solvent-accessible pathway connecting bulk solvent to the diiron catalytic core and whether D166 lies near its surface opening. Using MOLEonline analysis of the ScdA structure (PDB: 9j47), with the search seeded near the diiron center and a minimum bottleneck radius of 1.3 Å, we identified a single channel that connects the active-site region to the protein surface (Figure 3A).^17^ Along most of its length, the channel maintains a radius of ∼1.6 ± 0.3 Å and broadens to ∼2.3 Å near the surface, where only one branch is solvent-accessible. D166 is located adjacent to this surface-proximal opening, and its side chain can interact with S165, K135, and first-sphere H132 through hydrogen bonding or electrostatic contacts (Figure 3B). This geometry is consistent with a gatekeeping role in which the side-chain size and charge at position 166 modulate the effective aperture for nitrite and/or proton transfer.

**Figure 3:**
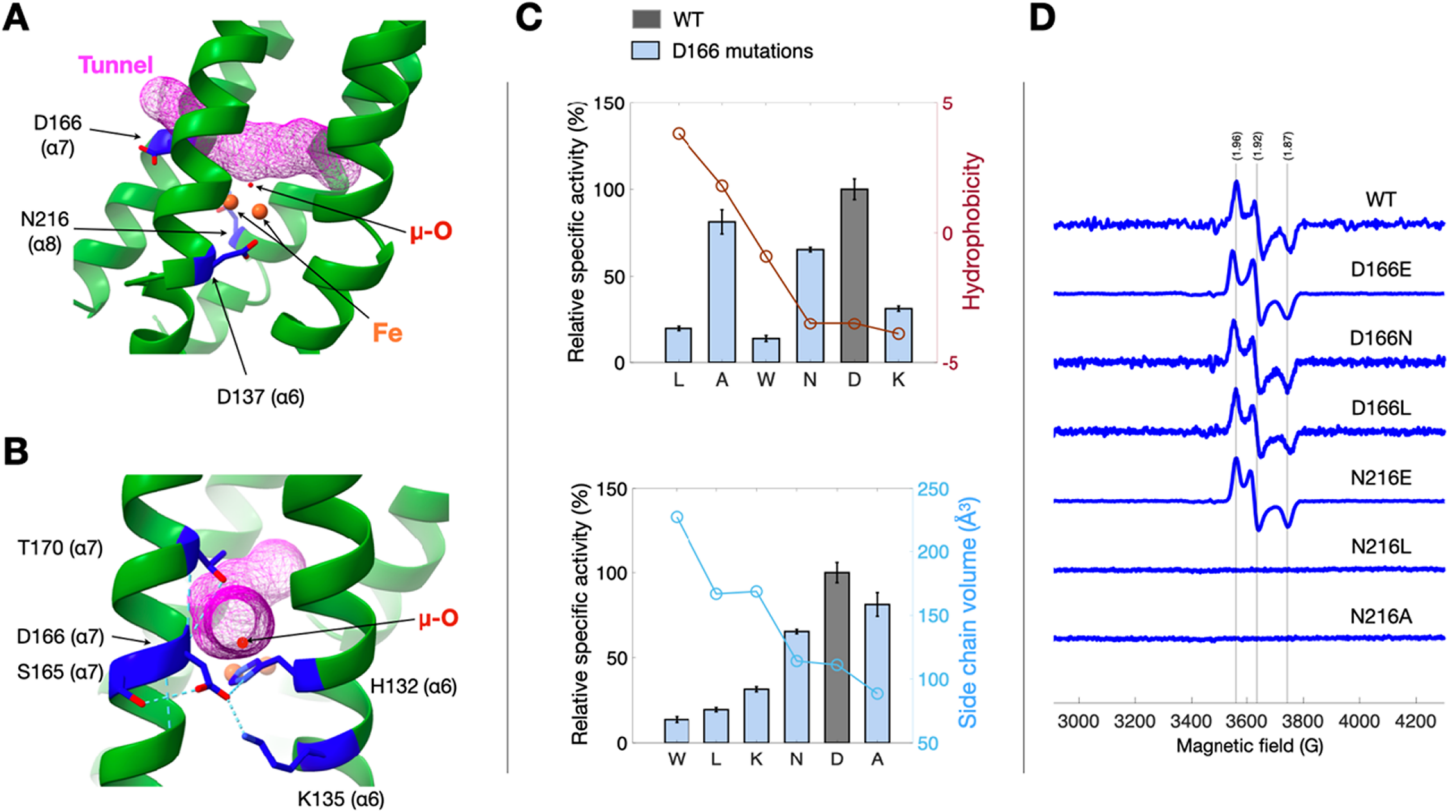
Structural and spectroscopic evidence supporting the tunnel-gating role of D166 in ScdA. (A) Tunnel analysis of ScdA (PDB 9j47) using MOLEonline reveals a solvent-accessible channel (magenta mesh) connecting the diiron active-site region to the protein surface, with the surface-proximal opening adjacent to D166. (B) Local interactions at the channel opening showing potential contacts between D166 and nearby residues S165, K135, and the first-sphere ligand H132. These interactions define a potential gating network controlling solvent entry to the tunnel. (C) Correlation of nitrite-reduction activity of D166 variants (blue bars) with physicochemical properties of their side chains. Top: relative activity versus hydrophobicity index; bottom: relative activity versus side-chain volume. WT (gray) is normalized to 100%. Data represent mean ± s.d. (*n* ≥ 3). (D) X-band EPR spectra (10 K) of WT and indicated variants showing the mixed-valent diiron signal (*g* ≈ 1.96, 1.92, 1.87) characteristic of the active cofactor. Mixed-valent populations were estimated by double integration of the mixed-valent region, with intensities normalized to protein concentration (relative spin counts, WT = 1.00): D166E = 1.27, D166N = 1.13, D166L = 1.15, N216E = 1.20. D166 variants (E, N, L) retain the mixed-valent signal, whereas N216L is EPR-silent, consistent with disruption of the cofactor environment. The reported spin counts quantify the relative mixed-valent population and are not used as proxies for catalytic activity, which is additionally influenced by second-sphere control of access/hydration and proton-transfer determinants.

Two complementary observations, informed by the analysis of the ScdA crystal structure (PDB: 9j47), support this model. First, extending the side chain at position 166 (D166E) plausibly strengthens a salt bridge between residue 166 and K135 observed in the structure, reorienting the local interaction network at the tunnel exit and enhancing solvent or substrate throughput; correspondingly, activity rises (∼1.5 × WT). Second, shortening the side chain (D166A) reduces steric occlusion at the same structural position while retaining ∼80% of the WT activity, consistent with partial relief of gating constraints. To assess whether steric effects dominate side-chain chemistry, we systematically examined D166W/L/K/N/A variants. Activity showed no clear dependence on hydrophobicity but increased monotonically as side-chain volume decreased (approximate size order W > L ≳ K > *N* > A; Figure 3C). Together, these structure-guided trends indicate that D166 functions primarily as a steric/electrostatic gate at the tunnel exit, modulating the flux to the diiron center without affecting metal occupancy.

EPR spectroscopy reinforces this division of labor. Variants D166E, D166N, and D166L retained the WT-like mixed-valent diiron signal at 10 K (Figure 3D), indicating that substitutions at 166 do not perturb the electronic structure of the cofactor. Nevertheless, the modest differences in mixed-valent spin counts (and small shifts in spectral parameters) are consistent with subtle second-sphere electrostatic perturbations at the diiron site, even though the mixed-valent state remains readily populated. In contrast, mutations that disrupt the putative electron-transfer hydrogen-bonding network at N216 altered the EPR spectral signature: N216E remains EPR-active, whereas the loss-of-function variants N216L and N216A are EPR-silent. These findings suggest that D166 primarily modulates substrate access through a surface-proximal gate, while residues such as N216 more directly influence the electron-transfer network linked to the diiron center.

### Solvent Kinetic Isotope Effects Implicate D166 in Access Gating

Because solvent access and proton transfer are often kinetically coupled in redox enzymes, solvent kinetic isotope effects (KIEs) provide a powerful means to distinguish whether turnover is limited by proton-dependent chemistry or by solvent-gated conformational or access steps. In particular, changes in the KIE magnitude and sign can reveal shifts in the rate-determining step arising from altered hydration dynamics near the catalytic core.

We therefore determined solvent kinetic isotope effects (KIE = *k*_H2O_/*k*_D2O_) for MV-coupled turnover in matched pH/pD buffers for the ScdA WT and selected second-sphere variants (Figure 4). ScdA WT exhibited KIE ≈ 1, indicating that under native conditions the rate-determining step does not involve transfer of a solvent-exchangeable proton. In contrast, substitutions at D166 produced divergent behavior: D166E displayed a mild inverse KIE (*k*_D2O_ > *k*_H2O_), whereas D166A showed a pronounced normal KIE (∼2-fold slower in D_2_O; KIE ≈ 2). The normal KIE observed for D166A suggests that a solvent-exchangeable proton-transfer step contributes to the rate-determining process, whereas the inverse KIE for D166E is indicative of a pre-equilibrium or access-limited step that is thermodynamically stabilized in D_2_O.

**Figure 4:**
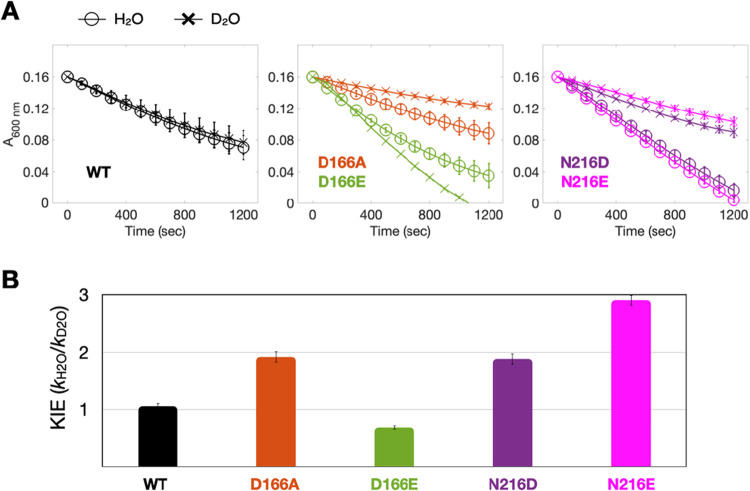
Solvent KIE distinguish solvent gating from electron-transfer perturbation in ScdA. (A) Time-dependent oxidation of MV cation radicals (A_600_ nm) by WT and selected second-sphere variants in matched H_2_O and D_2_O buffers. WT exhibits nearly identical reaction rates in both solvents. Substitutions at D166 produce divergent behavior: D166E shows a mild inverse isotope effect (*k*_D2O_ > *k*_H2O_), whereas D166A displays a pronounced normal isotope effect (∼2-fold slower in D_2_O). In contrast, variants at N216 (N216D and N216E) exhibit normal solvent isotope effects, indicating increased sensitivity to solvent-exchangeable proton transfer. (B) Quantified solvent kinetic isotope effects (KIE = *k*_H2O_/*k*_D2O_) derived from initial turnover slopes. The contrasting KIE signatures reveal distinct functional roles within the second coordination sphere: D166 modulates solvent accessibility and access gating, shifting the rate-determining step between access-limited and proton-transfer-sensitive regimes, whereas mutations at N216 are associated with normal solvent kinetic isotope effects, consistent with increased involvement of solvent-exchangeable proton transfer when electron-transfer efficiency is perturbed.

Notably, mutations at N216 yielded a distinct KIE profile. Both N216D and N216E exhibited normal solvent KIEs, with magnitudes comparable to or greater than those of D166A (Figure 4B). This behavior is consistent with increased involvement of solvent-exchangeable proton transfer when the electron-transfer network is perturbed, rather than with altered solvent access. The contrast between D166 and N216 variants thus reinforces a division of labor within the second coordination sphere: D166 primarily governs solvent accessibility and gating, whereas N216 modulates electron-transfer efficiency in a manner that indirectly enhances proton sensitivity.

Consistent with this interpretation, structural tunnel analysis (MOLEonline; Figure 3A) identifies a solvent-accessible pathway connecting the protein surface to the catalytic core through a D166-lined gate. This geometry positions D166 at a solvent-facing choke point, which is well suited to regulate solvent entry. Together, the KIE patterns and tunnel topology demonstrate that perturbations at D166 shift the rate-limiting step by modulating solvent access, whereas perturbations at N216 shift the reaction into a proton-transfer-sensitive regime without directly altering access pathways.

### MD Simulations Support Local, Not Global, Structural Effects

Because gating in a narrow tunnel could, in principle, be confounded by global instability, we next examined whether second-sphere substitutions distort the overall fold on a nanosecond time scale. To verify that our functional interpretations arise from local remodeling near the active site rather than wholesale structural rearrangements, we ran 150 ns MD simulations on WT ScdA and representative second-sphere variants (D137E/A, D166E/A, N216E/A). Trajectory convergence was assessed by backbone root-mean-square deviation (RMSD; Figure S3). All variants except D137A stabilized within ∼2 Å and retained the four-helix-bundle scaffold, confirming that single-site mutations at D166 and N216 introduce only localized effects. In contrast, D137A displayed markedly larger RMSD fluctuations and visible distortion of the C-terminal domain, consistent with its experimentally observed loss of cofactor integrity. These results confirm that mutation-induced functional differences primarily arise from local electrostatic or solvent-gating perturbations rather than from global unfolding.

### Cell-Based Spin-Trapping Links the *In Vitro* Mechanism to Cellular NO Production

Having defined how first- and second-sphere features shape catalysis *in vitro*, we asked whether these determinants govern NO formation inside cells. We monitored intracellular NO generation in *E. coli* overexpressing WT or mutant ScdA using a PTIO-based EPR assay.^16,18,19^ After nitrite addition (30 min), we introduced carboxy-PTIO, which reacts with NO to form PTI; PTIO and PTI exhibit distinct five- and seven-line EPR patterns, enabling spectral deconvolution (Figure 5A–D). WT showed clear PTI by 60 min after nitrite addition and continued increases at 75 and 90 min, indicating ongoing NO formation; catalytically competent second-sphere variants followed the same trend.

**Figure 5:**
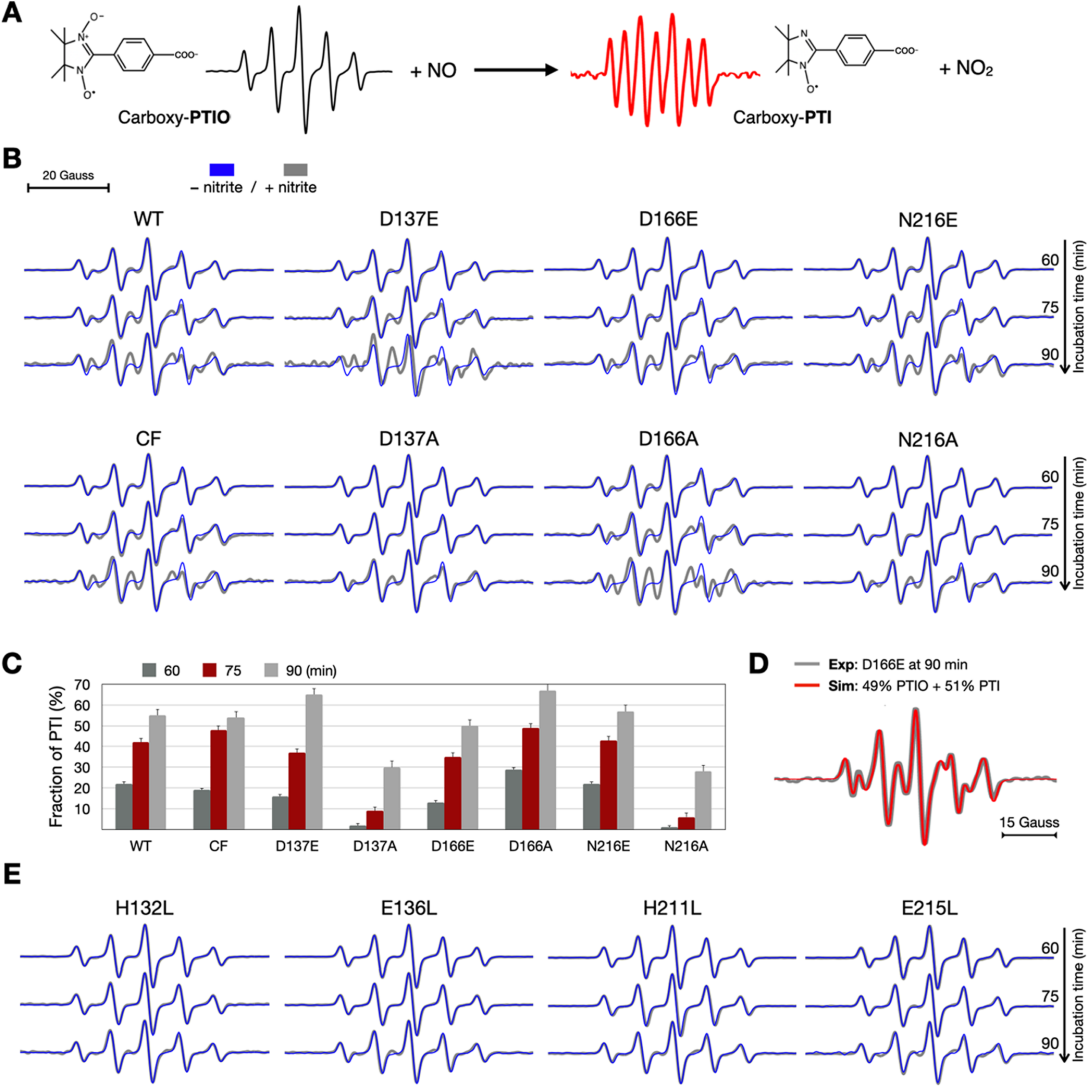
Cell-based EPR detection of NO generation catalyzed by ScdA variants. (A) Schematic of the PTIO-based spin-trapping assay. The membrane-permeable spin trap carboxy-PTIO reacts with NO to form carboxy-PTI, converting the characteristic PTIO EPR signal (black) to a distinct PTI signal (red). (B) Time-dependent X-band EPR spectra (*g* ≈ 2.0 region) of *E. coli* cells overexpressing ScdA variants recorded after incubation with nitrite for 60, 75, and 90 min. Blue and gray traces correspond to samples without and with nitrite, respectively. WT and catalytically competent variants (D137E, D166E, N216E) exhibit progressive PTI formation, whereas inactive mutants (D137A, N216A) show minimal change. The CF variant behaves similarly to WT. (C) Quantification of PTI fractions at 60, 75, and 90 min derived from linear combination fitting of PTIO and PTI spectra. Data represent mean ± s.d. (*n* ≥ 3). (D) Representative fit for D166E at 90 min showing excellent agreement between experimental (gray) and simulated (red) spectra, confirming reliable PTI quantitation. (E) EPR spectra of first-sphere mutants (H132L, E136L, H211L, E215L) showing no detectable PTI formation, consistent with loss of catalytic NO production. (see also Figure S4 for representative fits and ScdA expression results).

In contrast, loss-of-function variants D137A and N216A showed little or no PTI at 60 min and only minor PTI even at 75 min (<10%), mirroring their weak MV activities. Controls lacking nitrite remained dominated by PTIO throughout the time course (blue traces in Figure 5B), confirming that PTI formation in this assay is nitrite-dependent under our conditions. Importantly, upon nitrite addition, the early PTI accumulation is strongly variant-dependent, robust for WT and catalytically competent second-sphere variants but minimal for loss-of-function constructs—supporting a ScdA-dependent contribution to NO formation above a slower, host-derived background that becomes more apparent at later times.

To evaluate the potential involvement of native cysteines in the redox chemistry, we compared the WT to a cysteine-free construct (CF). WT and CF exhibited highly similar PTI time courses and magnitudes, indicating that the three native cysteine residues (C30, C31, and C191) are not directly engaged in the nitrite-to-NO electron-transfer step. These residues more likely modulate disulfide-linked oligomerization and thereby influence activity indirectly, consistent with prior observations.^11^

Finally, first-sphere substitutions (H132L, E136L, H211L, and E215L) produced essentially no PTI over 90 min (Figure 5E), reinforcing the essential role of intact first-sphere ligation for in-cell NO production and further validating the PTIO-based EPR assay as a faithful readout of ScdA activity in cells.

## Discussion

### An Integrated Mechanistic Model for ScdA Catalysis

Our results delineate how ScdA couples electron transfer with solvent-controlled substrate access at a nonheme diiron center (Figure 6). As expected from the established principles of diiron enzymology, the first coordination sphere provides an essential structural foundation for catalysis. Substitution of any histidine or glutamate ligand within the canonical 4-His/2-Glu framework abolishes metal incorporation and eliminates the characteristic mixed-valent EPR signal, confirming that the cofactor assembly and redox integrity strictly depend on intact first-sphere coordination. These observations establish a necessary mechanistic baseline against which more subtle regulatory effects can be interpreted.

**Figure 6:**
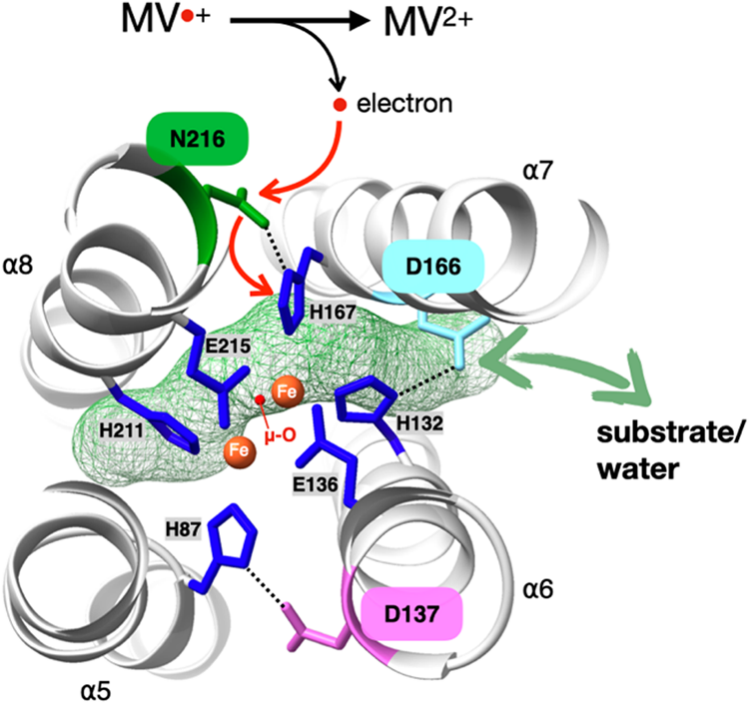
Proposed working model of ScdA catalysis. The diiron cofactor (orange spheres) is coordinated by first-sphere residues (dark blue). N216 mediates electron transfer from the external donor (MV^•+^ → MV^2+^), while D166 gates substrate and solvent access through a tunnel connecting the μ-oxo bridge to the protein surface. D137 contributes to stabilizing the diiron coordination. The figure illustrates the mechanistic roles of N216 and D166 in regulating electron delivery and substrate/hydration dynamics at the active site.

Surrounding this structural core, second-sphere residues exert distinct and finely tuned control over the catalytic turnover. In contrast to the binary loss-of-function behavior observed for first-sphere substitutions, single-site mutations in the second sphere produce graded effects on activity, while largely preserving iron loading. Variants that maintain hydrogen-bonding capacity or favorable electrostatic interactions retain moderate activity, whereas the removal of donors or increases in steric bulk markedly diminish turnover. These trends indicate that second-sphere interactions stabilize the geometry and protonation state of the first-sphere ligands and thereby modulate the electron-transfer efficiency rather than the cofactor integrity.

Within this regulatory network, residues at N216 and D166 play separable yet complementary roles in controlling diiron-center catalysis. Substitutions at N216 perturb a hydrogen-bonding network linked to electron delivery to the diiron center, shifting turnover into a proton-transfer-sensitive regime. In contrast, substitutions at D166 define a solvent-accessible gate that regulates the flux of water and substrate to the catalytic core, thereby modulating local hydration dynamics and shifting the rate-limiting step between access-limited and proton-transfer-limited regimes. Consistent with an access- and substrate-dependent component to WT turnover, our prior Michaelis–Menten analysis showed that ScdA activity varies systematically with nitrite concentration.^11^ These local perturbations do not alter the global fold of ScdA, as supported by MD simulations, but instead fine-tune catalytic competence through distinct second-sphere mechanisms. Together, these findings illustrate how the protein matrix coordinates electron transfer and solvent accessibility to regulate diiron-center reactivity, extending established paradigms of second-sphere redox modulation—previously defined largely for oxidative metalloenzymes—to a reductive transformation and highlighting solvent gating and hydration control as general design principles in nonheme diiron enzymology.^20–22^

### Solvent Gating, KIE Behavior, and Hydration Dynamics

The solvent KIE results (Figure 4), together with structural analysis, clarify how solvent accessibility influences ScdA catalysis and help rationalize why perturbations at D166 affect activity. Wild-type ScdA exhibits a near-unity KIE, indicating that under native gating conditions, the rate-determining step does not involve transfer of a solvent-exchangeable proton. In contrast, variant D166E displays a mild inverse KIE, consistent with a gating or pre-equilibrium step stabilized in D_2_O, whereas variant D166A shows a pronounced normal KIE (∼2), indicating that a solvent-exchangeable proton-transfer step becomes rate-limiting when solvent access is increased. These contrasting behaviors show that modest changes at the tunnel exit can shift the catalytic bottleneck between access-limited and proton-transfer-limited regimes.

Structural mapping using MOLEonline identifies a solvent-accessible tunnel connecting the diiron center to the protein surface through a D166-lined gate, providing a plausible route for transient entry of water into the catalytic core. In nonheme diiron systems, bridging oxygen species and associated hydrogen-bond networks are known to be sensitive to local hydration, and solvent substitution (H_2_O → D_2_O) can perturb these equilibria through changes in hydrogen-bond strength and p*K*_a_ values. Experimental studies on diiron model complexes and enzymes have shown that protonation or hydration at bridging sites can influence electronic coupling and redox behavior.^23–25^

To complement the static tunnel model with a trajectory-based measure of hydration, we quantified local water occupancy around residues that frame the surface-proximal opening of the mapped pathway (G128, M129, H132, position 166, A169, and T170) across MD trajectories (Figure S5). Water occupancy (*N*w) was computed as the number of water molecules within 3.5 Å of each residue per frame and is summarized as probability distributions. The summed water occupancy across this six-residue set is similar for WT and D166E (Σ*N*w ≈ 16.5), whereas D166A shows a modest decrease (Σ*N*w ≈ 14.5). Importantly, D166A produces a pronounced redistribution of hydration within the entrance region: water occupancy at position 166 decreases substantially (WT ∼ 7.3 → D166A ∼ 1.6), while neighboring lining residues exhibit increased hydration, including M129 (2.7 → 4.7), H132 (0.28 → 1.2), and T170 (1.89 → 2.6). Thus, although total hydration near the entrance changes only modestly, the “open-gate” D166A substitution shifts water association away from the side chain at position 166 and toward adjacent residues lining the access corridor. These MD results provide direct, solution-phase support that substitutions at D166 remodel the local hydration landscape at the pathway opening, consistent with the KIE-derived shifts between access-limited and proton-transfer-sensitive regimes.

Within this framework, the KIE pattern observed for ScdA (unity for the WT, inverse for D166E, and normal for D166A) supports a model in which D166 modulates solvent access and thereby influences which elementary step limits turnover. In the D166E variant, modest tightening or electrostatic reorganization of the gate likely restricts solvent entry sufficiently to shift the rate limitation toward a gating or pre-equilibrium step (inverse KIE), correlating with enhanced activity. In contrast, reduced steric constraint in D166A increases solvent throughput, rendering a downstream solvent-exchangeable proton-transfer step rate-limiting (normal KIE) and diminishing turnover. Similar sensitivity of catalytic kinetics to hydration state has been noted in other nonheme diiron enzymes, including soluble methane monooxygenase hydroxylase and AurF.^9,26,27^

Collectively, these observations indicate that solvent access through the D166-lined tunnel must be regulated rather than maximized to support efficient catalysis. ScdA thus represents a reductive system in which controlled hydration and solvent gating contribute to tuning the diiron-center reactivity, extending concepts previously developed for oxidative diiron enzymes to nitrite reduction.

### Physiological Relevance and Implications

Finally, the cell-based NO production assay in *E. coli* confirms that these structural and mechanistic features are physiologically meaningful rather than artifacts of *in vitro* reconstitution. Cells expressing active ScdA generate NO upon nitrite exposure, whereas variants impaired in redox coupling or solvent gating do not. Although methyl viologen is an artificial electron donor, the same structural constraints are expected to govern electron transfer from native partners, underscoring the generality of this gating model. Likewise, in our heterologous *E. coli* expression system, ScdA is unlikely to engage its native redox partner(s), so the cell-based assay is intended to test whether the same structural determinants remain operative in living cells rather than to replicate the native electron-delivery pathway.

Taken together, our findings identify solvent gating and hydration control as central design elements in nonheme diiron catalysis. ScdA exemplifies how a balanced network of first- and second-sphere interactions coordinates electron delivery, proton management, and substrate access within a compact protein matrix. This study offers a mechanistic framework for nitrite reduction and a broader lens on how dynamic hydration and hydrogen-bond rearrangements regulate the redox chemistry across the diiron enzyme family.

## Conclusion

This study defines a unifying mechanistic framework for nitrite reduction by the nonheme diiron enzyme ScdA. Through integrated structural, spectroscopic, kinetic, and computational analyses, we show that catalytic efficiency arises from the concerted action of first-sphere ligands that ensure cofactor integrity and second-sphere interactions that regulate electron delivery and solvent accessibility. The discovery that controlled hydration near the catalytic core modulates activity establishes solvent gating as a general strategy for tuning the redox reactivity in diiron centers. By demonstrating that the same structural determinants controlling catalysis *in vitro* also govern NO formation in cells, this work directly links chemical mechanism to physiological function. These findings not only clarify how ScdA achieves efficient electron–proton coordination during NO formation but also illustrate how protein matrices harness solvent dynamics to govern catalysis across the nonheme diiron enzyme family.

## Materials and Methods

### Protein Sample Preparation

The full-length *S. aureus* scdA gene was cloned into the NcoI/*Eco*RI sites of pET-28a (New England Biolabs).^11^ Site-directed mutagenesis (QuikChange, Stratagene) generated single-point variants using the wild-type (WT) construct as a template. A cysteine-free (CF) variant (C30A-C31A-C191A) was also prepared to eliminate native cysteines. All mutations were confirmed by DNA sequencing and expressed in *E. coli* BL21­(DE3) cells (Agilent).

Unless otherwise noted, recombinant proteins carried an N-terminal His_6_-tag.^11^ Cultures were grown overnight in LB (40 μg/mL kanamycin) at 37 °C, diluted 1:10 into fresh medium, and induced at OD_600_ ≈ 1.0 with 0.2 mM FeCl_3_ and 0.4 mM IPTG for 6 h at 30 °C. Cells were harvested by centrifugation and stored at −80 °C.

Cell pellets were lysed in 20 mM Tris and 150 mM NaCl (pH 7.6) with 0.6 mM PMSF by 10 min sonication on ice. After centrifugation (23,000*g*, 50 min) and filtration, supernatants were purified on a HisTrap HP Ni^2+^-affinity column, washed with 45 mM imidazole, and eluted with 300 mM imidazole. Imidazole was removed by PD-10 desalting into a storage buffer (20 mM Tris, 150 mM NaCl, 10% v/v glycerol, pH 7.6). Protein purity and oligomeric state were verified by SDS-PAGE and gel filtration (Superdex 75 Increase 10/300 GL). Protein concentrations were determined by UV–vis absorption at 280 nm (with an extinction coefficient of 40,800 M^–1^ cm^–1^).

### Nitrite Reductase Assay

Reduced methyl viologen (MV) was prepared following a reported photochemical protocol under strictly anaerobic conditions.^28^ Briefly, 4 mg of MV was dissolved in 1 mL of ethanol and irradiated at 365 nm until the solution turned deep blue. The solvent was evaporated, and the residue was resuspended in assay buffer to yield reduced MV. For kinetic measurements, 1 μM ScdA was mixed with 116 μM reduced MV in an anaerobic cuvette, and the reaction was initiated by adding NaNO_2_ (final concentration: 3 μM from a 100 μM stock).^11^ MV oxidation was monitored by a decrease in absorbance at 600 nm (with an extinction coefficient of 13.7 mM^–1^ cm^–1^) over 1200 s. Initial rates were obtained from the linear portion of the trace and plotted as a function of nitrite concentration to assess catalytic activity.

### Iron Quantification by TPTZ Assay

Iron content in ScdA and its variants was quantified using the iron chelator 2,4,6-Tripyridyl-1,2,3-triazine (TPTZ).^16,29^ As-isolated ScdA protein (8 nmol) was incubated in 75 μL of 3 M HCl at 60 °C for 1 h to denature the protein and release bound iron. The solution was then mixed with 100 μL of 1 mM TPTZ and 10 μL of 10 mM sodium dithionite, followed by pH adjustment to 4.0 using 1 M sodium acetate. The total reaction volume was increased to 300 μL. The resulting Fe­(II)–TPTZ complex exhibited a characteristic absorbance maximum at 595 nm, which was measured by UV–visible spectroscopy. Iron concentrations were determined from a calibration curve generated using standard Fe­(II) solutions treated under identical conditions.

### Continuous Wave (CW) EPR Measurements

EPR spectroscopy was performed to determine the oxidation states of the diiron center in various as-isolated ScdA variants. Final protein concentrations of around 0.2 mM were loaded into 20 μL of sealed quartz capillaries under anaerobic conditions. Measurements were conducted at 10 K on a Bruker ELEXSYS 580 system equipped with an X-band microwave bridge, an ER 4122 SHQE resonator, and a cryogen-free Stinger cooling system. Standard parameters included a microwave frequency of approximately 9.7 GHz, a modulation amplitude of 1 G, a 100 kHz modulation frequency, and an incident microwave power of 1.5 mW. These low-temperature, low-power conditions minimized signal saturation and enhanced the resolution of mixed-valence or high-spin states.

### Assessment of In-Cell Nitric Oxide (NO) Production Using Spin Trapping

*E. coli* BL21­(DE3) cells harboring the ScdA expression plasmid were grown aerobically in 50 mL of LB medium containing kanamycin (40 μg mL^–1^) at 37 °C overnight. The cultures were harvested by centrifugation and resuspended in fresh LB (50 mL) supplemented with 0.2 mM FeCl_3_, followed by incubation at 37 °C for 1 h to promote iron uptake. Protein expression was induced by adding 0.4 mM IPTG, and the cells were further incubated at 30 °C for 6 h.

For in-cell detection of NO, 1 mL aliquots of the induced *E. coli* cultures were incubated with or without 50 mM NaNO_2_ at 37 °C for 30 min. Carboxy-PTIO (2-phenyl-4,4,5,5-tetramethylimidazoline-1-yloxyl-3-oxide; 100 μM final concentration) was then added, and incubation continued for another 30 min under identical conditions.^16,18,19^ Upon reaction with NO, PTIO is converted to the nitroxide product PTI, resulting in a characteristic change in the EPR spectrum—from a five-line to a seven-line pattern, reflecting NO trapping and spin-adduct formation. Before EPR measurements, 1.5 mM potassium ferricyanide was added to oxidize any reduced radical species, and 20 μL of the suspension was transferred to sealed glass capillary tubes. Control samples lacking nitrite were prepared in parallel.

Cell-based EPR spectra were recorded on a Bruker Magnettech ESR5000 spectrometer operating at an X-band frequency (9.4 GHz) under the following conditions: 100 kHz modulation frequency, 2 G modulation amplitude, 150 G sweep width, 1024 data points, one scan, and microwave power of 1.0 mW. All measurements were performed at 298 K. The relative populations of carboxy-PTIO and its NO-derived product, carboxy-PTI, were quantified from the EPR spectra to assess NO formation in *E. coli* cells expressing ScdA and its variants.

### Molecular Dynamics Simulations

Molecular dynamics (MD) simulations were conducted using OpenMM with the CHARMM36 force field.^30^ The starting structure was the *S. aureus* ScdA C-terminal domain (residues 67–224; PDB 9j47). Because robust classical force-field parametrization of nonheme diiron sites is system-specific and can introduce model-dependent artifacts, we focused the MD analysis on scaffold stability using an apo-like representation with restrained first-sphere geometry. To isolate mutation-induced effects on the protein scaffold (independent of metal-loading differences observed experimentally), the diiron center was omitted and harmonic restraints (*k* = 100 kJ mol^–1^ nm^–2^) were applied to the six first-sphere residues to preserve the crystallographic coordination geometry. Each protein was solvated in a TIP3P water box with Na^+^/Cl^–^ ions at a 150 mM ionic strength. Following 5000 steps of energy minimization, systems were heated gradually to 300 K under NPT conditions using Langevin dynamics. Long-range electrostatics were treated with the particle mesh Ewald method and periodic boundary conditions. Hydrogen-bond constraints were enforced via SHAKE with a 2 fs integration time step. Each system underwent 3 × 10^6^ restrained equilibration steps, followed by an unrestrained 150 ns production run. All simulations were performed in triplicate to ensure reproducibility and trajectory convergence. Trajectories were analyzed with MDTraj, and backbone stability was quantified using Cα RMSD values.^31^ In addition, local hydration was quantified by analyzing the MD trajectories with MDAnalysis to compute water occupancy (water counts *N*w) within a specified cutoff distance (3.5 Å) of selected residues over time (Figure S5). The 150 ns duration and replicate design provided statistically robust sampling of conformational fluctuations among the ScdA variants. Molecular graphics and analyses were performed with UCSF ChimeraX.^32^

## Supplementary Material



## Notes

Experimental data for the MV assays (Figure S1); representative experimental data for the TPTZ assays (Figure S2); MD simulations result (Figure S3); representative fits of EPR spectra (Figure S4); and MD-derived water occupancy mapping (Figure S5) (PDF)
