# Supplementary material for: Matrix Control of Solvent and Electron Flow in a Nonheme Diiron Nitrite Reductase
Source: JACS Au. 2026 Mar 26;6(4):2445–54. doi: 10.1021/jacsau.6c00043 (PMC13126187; doi:10.1021/jacsau.6c00043)
Supplement: Supplementary file 1 [file au6c00043_si_001.pdf]

Supporting Information for

Matrix Control of Solvent and Electron Flow in a Non-Heme Diiron Nitrite Reductase

Hung-Ying Chen,[†] Yi-Shan Lu,[†] Chu-Chun Cheng, Feng-Chun Lo, Tzuhsiung Yang, and Yun-Wei Chiang*

Department of Chemistry, National Tsing Hua University, Hsinchu, Taiwan

*Corresponding email: ywchiang@mx.nthu.edu.tw

[†]These authors contributed equally to this work.

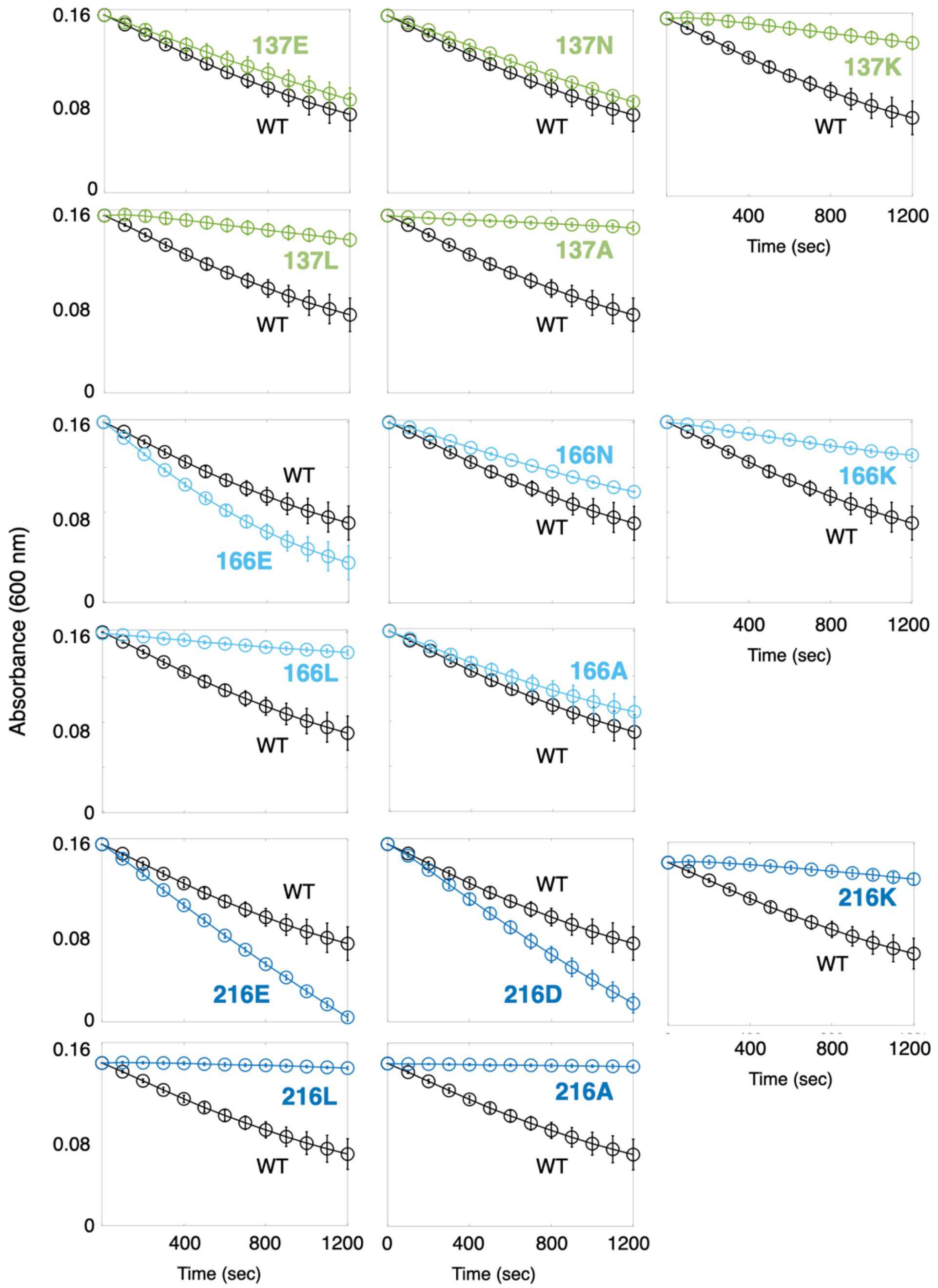

Figure S1. Comparison of MV-based nitrite reduction activities for second-sphere ScdA variants.

Time-dependent absorbance changes at 600 nm (A_{600}) reflect oxidation of reduced methyl viologen (MV) during nitrite reduction by ScdA. Each panel shows the reaction trace for wild-type (WT, black) and one variant (colored). Mutations were introduced at second-sphere residues D137

(green), D166 (cyan), and N216 (blue). Reactions contained 1 μM ScdA, 116 μM reduced MV, and nitrite under strictly anaerobic conditions. Linear fits to the initial decay phase were used to calculate initial rates. Variants that retained hydrogen-bonding capability (D137E, D137N, D166E, N216E) exhibited activities near or even greater than WT, whereas substitutions that disrupted hydrogen bonding or altered side-chain volume (e.g., D137A, D166K, N216L, N216A) significantly decreased MV oxidation. Data are representative of three independent measurements; error bars denote standard deviations (n = 3).

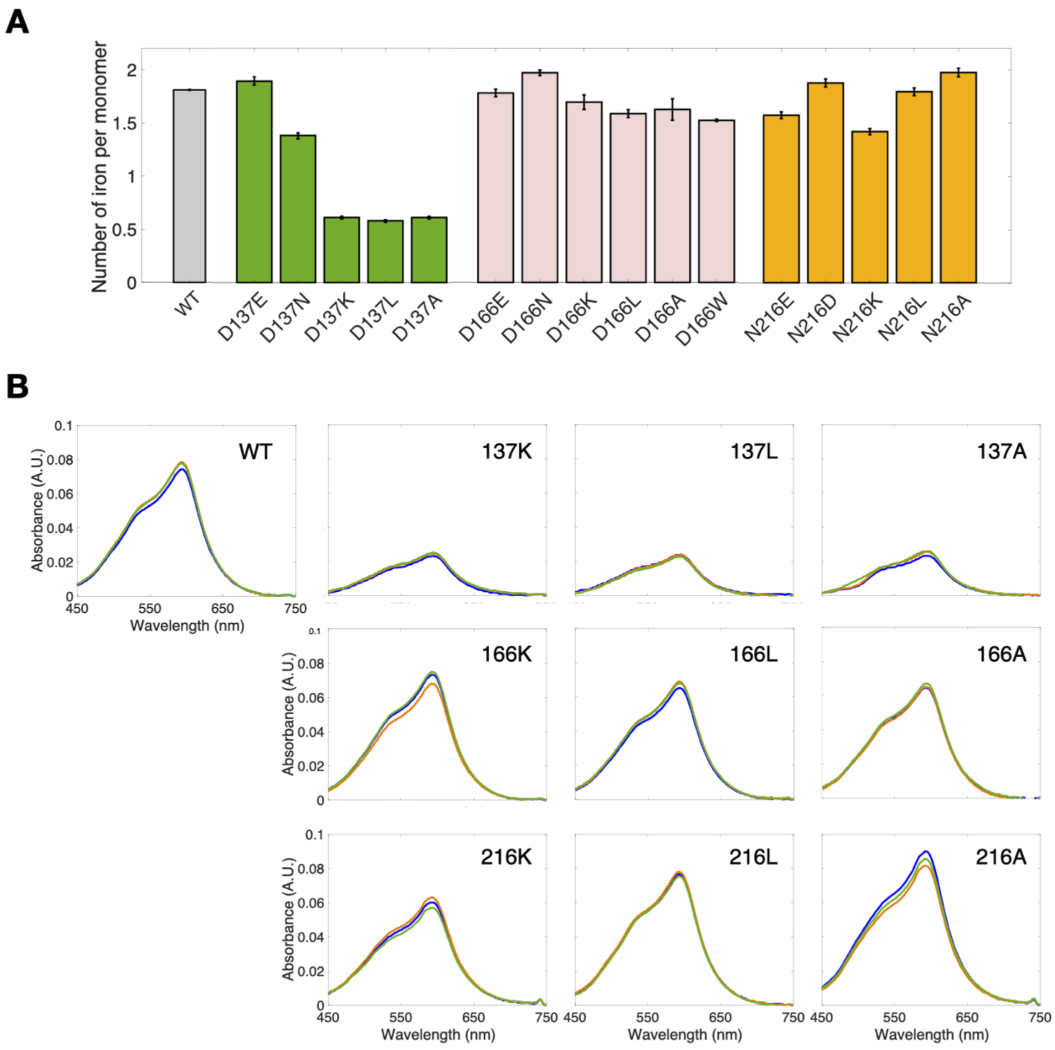

Figure S2. Iron quantification and representative TPTZ spectra of ScdA variants.

(**A**) Iron content per ScdA monomer, determined by the TPTZ colorimetric assay. Each bar represents the mean ± standard deviation from three independent measurements (n = 3). Variants at the D137 (green), D166 (pink), and N216 (orange) positions were analyzed alongside WT (gray). Mutations that disrupt hydrogen bonding or alter side-chain polarity at D137 substantially reduced iron incorporation, whereas D166 and N216 variants retained near-wild-type metal loading.

(**B**) Representative UV–Vis absorption spectra (450–750 nm) of the TPTZ–Fe^{2+} complex measured for WT and selected variants. Reduced absorbance at ~595 nm corresponds to lower iron content in specific mutants, consistent with quantitative data in panel A.

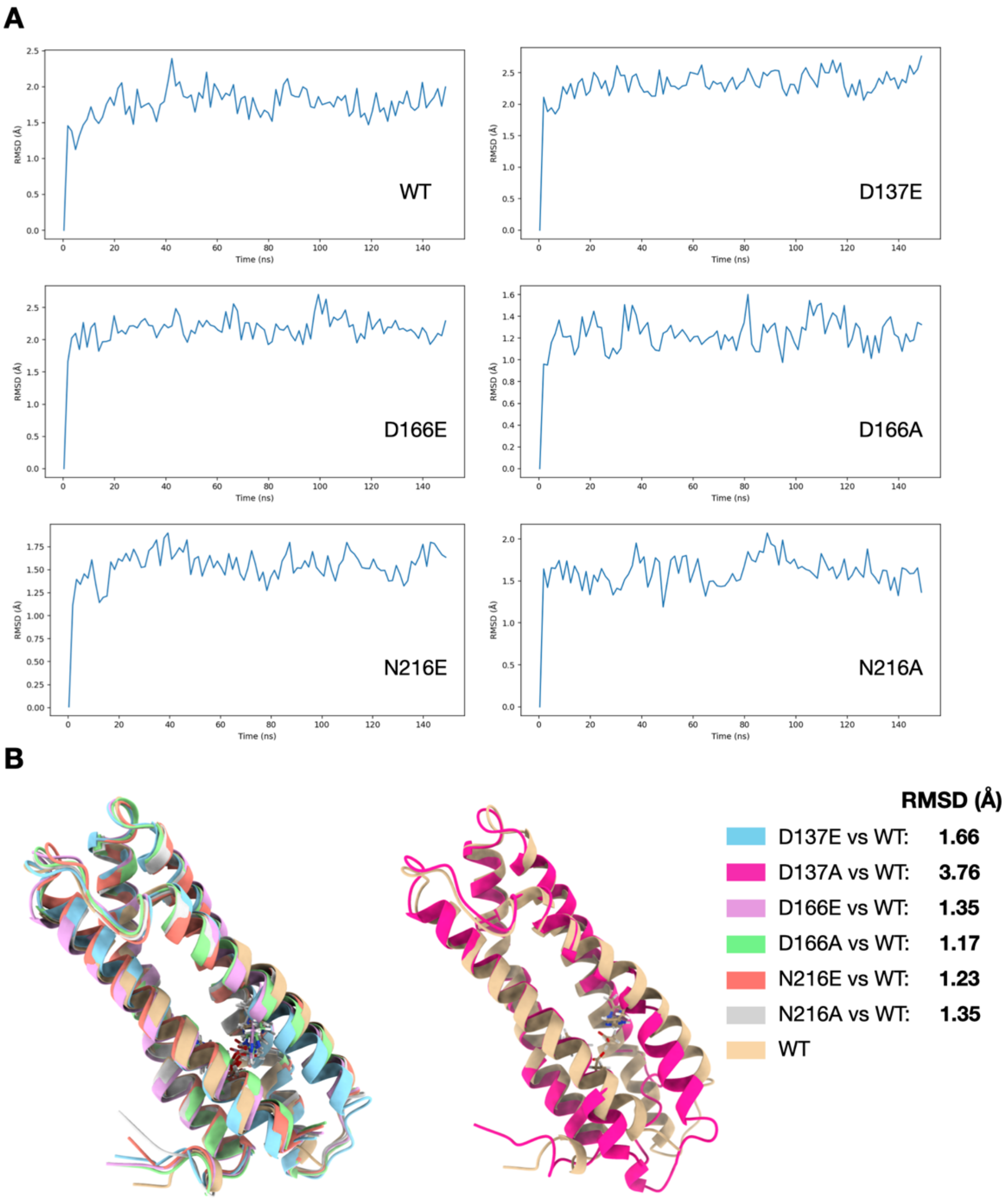

Figure S3. MD simulations reveal structural stability of ScdA variants.

(**A**) Time evolution of Cα root-mean-square deviation (RMSD) over 150-ns MD simulations for WT and selected variants (D137E, D166E, D166A, N216E, N216A). All trajectories show rapid equilibration followed by stable RMSD plateaus, indicating convergence and preservation of the overall fold on the simulation timescale.

(B) Superposition of final MD structures relative to WT. Variants D137E, D166E, D166A, N216E, and N216A (colored) align closely with WT (wheat), with pairwise RMSD values ranging from 1.17 Å to 1.66 Å. In contrast, D137A (magenta) shows a substantially larger deviation (RMSD =

3.76 Å), consistent with experimental evidence that perturbation at this position compromises cofactor-related structural integrity. Collectively, these data indicate that—aside from D137A—single-site substitutions do not measurably disrupt the CTD fold, supporting the interpretation that the observed functional differences arise from localized effects rather than global structural destabilization. Hydrogen-bond occupancy analysis was performed with the geometric cut-offs (donor–acceptor distance ≤ 3.0 Å; donor–hydrogen–acceptor angle ≥ 150°). In WT, the analysis indicates that the H167–N216 interaction is highly populated (~82%), whereas the H132–D166 interaction is less frequent (~22%), consistent with a more persistent hydrogen-bonding network associated with N216.

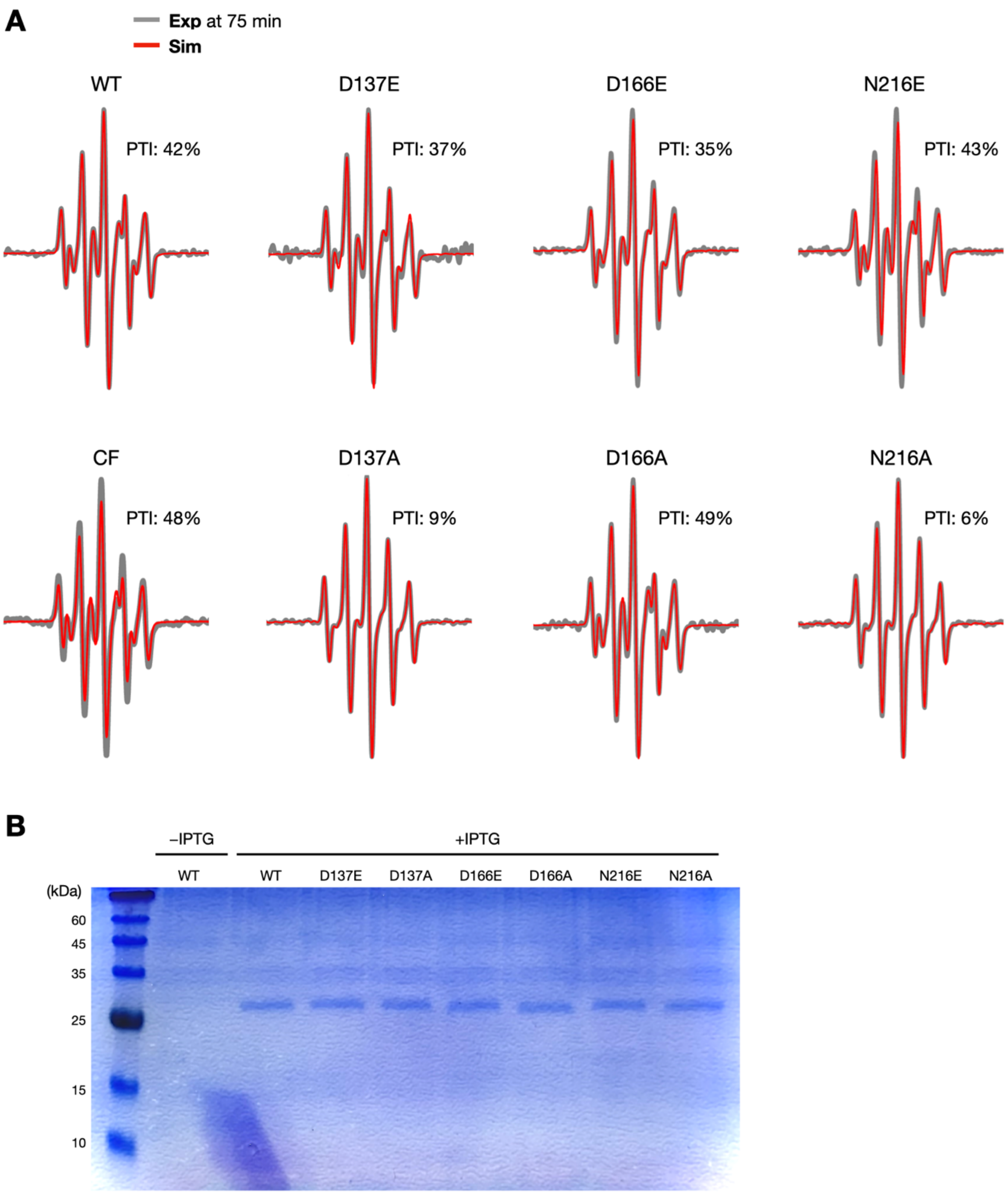

Figure S4. Representative spectral fits and SDS-PAGE verification of ScdA expression for cell-based NO detection.

(**A**) Representative EPR spectra recorded 75 min after nitrite addition showing excellent agreement between the experimental spectrum (gray) and the linear-combination fit (red) generated from PTIO and PTI reference spectra. The resulting PTI fractions are reported in the plots and summarized in Figure 5C. Spectra are displayed over an 80 G field window.

(**B**) SDS-PAGE analysis of lysates from E. coli cultures used for the cell-based PTIO spin-trapping assay, confirming comparable expression levels of ScdA WT and the indicated second-sphere variants.

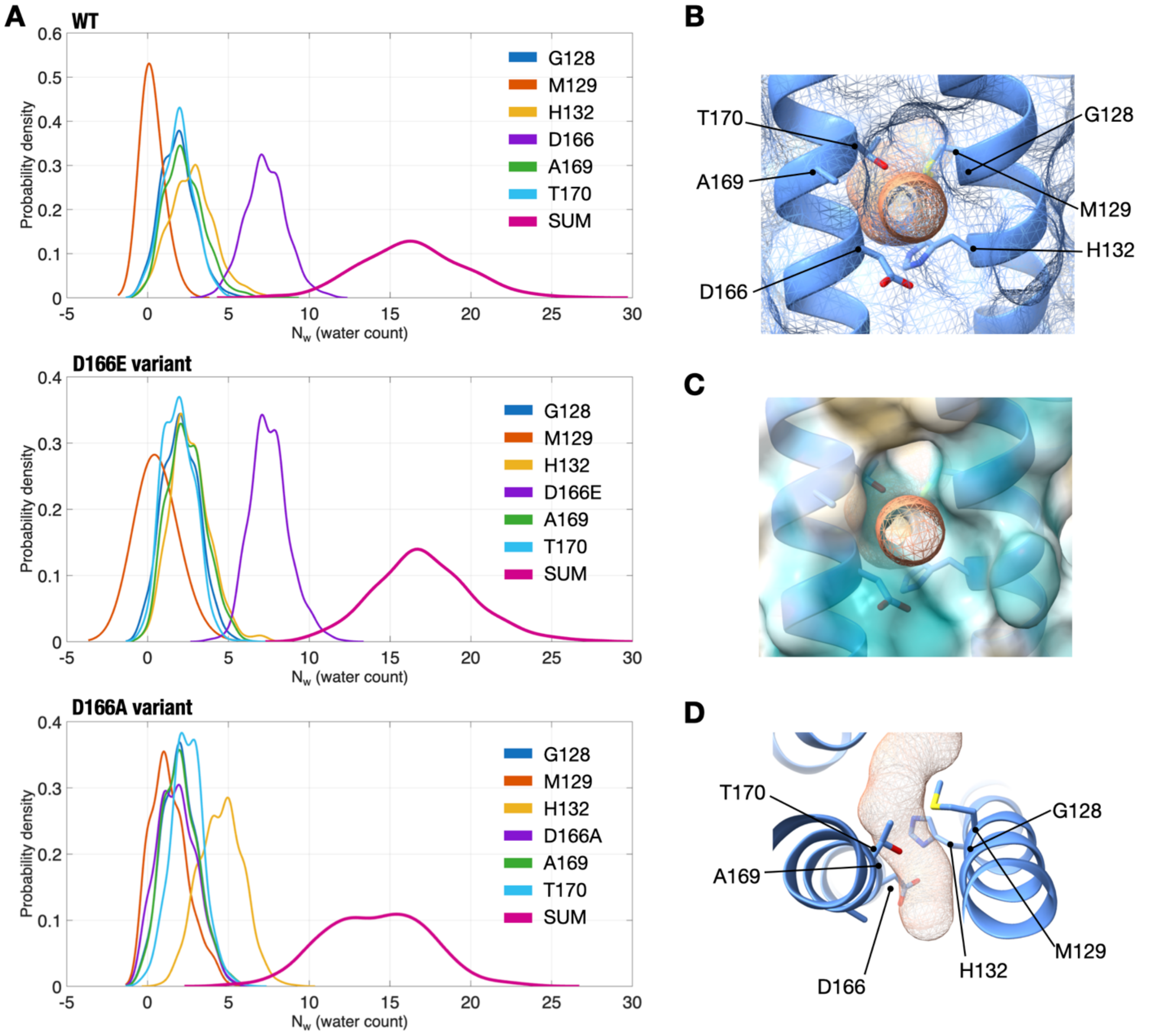

Figure S5. MD-derived water-occupancy mapping and local surface properties at the surface-proximal opening of the putative access pathway in ScdA.

(**A**) Probability distributions of water occupancy (*N*w; “water count”) for residues surrounding the surface-proximal opening of the pathway—G128, M129, H132, position 166 (D166/D166E/D166A), A169, and T170—calculated from MD trajectories of WT ScdA (top), D166E (middle), and D166A (bottom). For each residue, *N*w was computed frame-by-frame as the number of water molecules within 3.5 Å of the residue, and the resulting values were converted to probability density functions. Colored traces correspond to individual residues; the magenta trace (“SUM”) represents the instantaneous *N*w summed over all six residues, providing an aggregate measure of hydration in the entrance region over the trajectory. Across WT and D166E,

the summed hydration is similar ($\Sigma N\text{w} \approx 16.5$ for both), indicating comparable overall water occupancy at the entrance region in these two constructs. In contrast, D166A shows a modest reduction in the summed water count ($\Sigma N\text{w} \approx 14.5$) but a pronounced redistribution of hydration among the lining residues. Specifically, D166A markedly reduces local water occupancy at position 166 ($N\text{w} \approx 7.3$ in WT → 1.6 in D166A), consistent with replacement of a charged, solvent-engaging side chain by alanine. Importantly, this decrease is accompanied by substantial increases in water occupancy at neighboring residues that line the entrance corridor, including M129 ($N\text{w} \approx$ 2.7 in WT → 4.7 in D166A), H132 ($N\text{w} \approx 0.28$ → 1.2), and T170 ($N\text{w} \approx 1.89$ → 2.6). By comparison, these residues show only minor changes between WT and D166E. Thus, although the total hydration of the entrance residue set changes only modestly, the D166A substitution shifts water association away from the gate residue itself and toward other residues that extend toward the interior of the mapped pathway. This trajectory-based redistribution is consistent with the idea that side-chain identity at position 166 helps shape the local hydration environment at the surface opening and can influence how readily water molecules populate the access corridor.

(**B**) Structural rendering illustrating the locations of the six residues (sticks) defining the surface-proximal region of the pathway, as suggested by MOLEonline mapping. The channel volume is shown as a mesh to visualize connectivity and the proximity of position 166 to the surface opening.

(**C**) Surface-property visualization of the region surrounding the opening, colored by local hydrophilic/hydrophobic character (cyan, more hydrophilic; yellow, more hydrophobic), highlighting the predominantly hydrophilic character of the protein surface near the opening.

(**D**) Alternate view of the mapped pathway and surrounding secondary-structure elements, emphasizing the spatial arrangement of the six residues relative to the surface-proximal opening.
